# Corneal Regeneration by Deep Anterior Lamellar Keratoplasty (DALK) Using Decellularized Corneal Matrix

**DOI:** 10.1371/journal.pone.0131989

**Published:** 2015-07-10

**Authors:** Yoshihide Hashimoto, Seiichi Funamoto, Shuji Sasaki, Jun Negishi, Takako Honda, Shinya Hattori, Kwangwoo Nam, Tsuyoshi Kimura, Manabu Mochizuki, Hisatoshi Kobayashi, Akio Kishida

**Affiliations:** 1 Institute of Biomaterials and Bioengineering, Tokyo Medical and Dental University, Tokyo, Japan; 2 Department of Ophthalmology, Tokyo Medical and Dental University, Tokyo, Japan; 3 International Center for Materials Nanoarchitectonics, National Institute for Materials Science, Tsukuba, Japan; Institute for Frontier Medical Sciences, Kyoto University, JAPAN

## Abstract

The purpose of this study is to demonstrate the feasibility of DALK using a decellularized corneal matrix obtained by HHP methodology. Porcine corneas were hydrostatically pressurized at 980 MPa at 10°C for 10 minutes to destroy the cells, followed by washing with EGM-2 medium to remove the cell debris. The HHP-treated corneas were stained with H-E to assess the efficacy of decellularization. The decellularized corneal matrix of 300 μm thickness and 6.0 mm diameter was transplanted onto a 6.0 mm diameter keratectomy wound. The time course of regeneration on the decellularized corneal matrix was evaluated by haze grading score, fluorescein staining, and immunohistochemistry. H-E staining revealed that no cell nuclei were observed in the decellularized corneal matrix. The decellularized corneal matrices were opaque immediately after transplantation, but became completely transparent after 4 months. Fluorescein staining revealed that initial migration of epithelial cells over the grafts was slow, taking 3 months to completely cover the implant. Histological sections revealed that the implanted decellularized corneal matrix was completely integrated with the receptive rabbit cornea, and keratocytes infiltrated into the decellularized corneal matrix 6 months after transplantation. No inflammatory cells such as macrophages, or neovascularization, were observed during the implantation period. The decellularized corneal matrix improved corneal transparency, and remodelled the graft after being transplanted, demonstrating that the matrix obtained by HHP was a useful graft for corneal tissue regeneration.

## Introduction

Corneal transplantation (penetrating keratoplasty, PKP) is the only effective therapy for many disorders of the cornea that can lead to visual impairment and blindness [1]. Nevertheless, immune-mediated corneal allograft rejection remains a major cause of graft failure. A recent study reported that the cumulative incidence of rejection episodes was 23.2%, and the cumulative incidence of irreversible rejection was 5.2% in eyes after treatment with PKP [2]. In addition, continuous decrease of corneal endothelium is a major problem after PKP [3], and lamellar surgical techniques (lamellar keratoplasty, LKP) have been developed to only remove damaged tissue.

Deep anterior lamellar keratoplasty (DALK) has a number of advantages over PKP. This technique replaces the recipient corneal epithelium and stroma by donor cornea, whereas the recipient corneal endothelium and Descemet’s membrane remain [4]. Thus, there is no endothelial rejection and no need for a corneal graft with normal endothelial function. DALK has therefore recently become an alternative surgical approach for patients with a hazy cornea, keratoconus, trauma, stromal dystrophies, stromal scars after infectious keratitis, and corneal thinning with an intact corneal endothelial function [4–9]. However, cellular components of the corneal graft, including the epithelium and keratocytes, can cause epithelial and stromal rejections.

One way to overcome these drawbacks is to use a decellularized corneal matrix, from which the major immunogenic cellular components, including lipid membranes and membrane-associated antigens, are removed to reduce the immune rejection, while still maintaining the integrity of the extracellular matrix (ECM). Decellularized corneal matrix is therefore promising as an ideal scaffold for corneal tissue regeneration and corneal tissue engineering. Several decellularization techniques using chemical, enzymatic, and physical treatments have been proposed for preparing decellularized corneal matrices [10–11]. In general, chemical decellularization uses a detergent such as sodium dodecyl sulfate (SDS), polyoxyethylene octylphenyl ether (TritonX-100), 3-[(3-cholamidopropyl)dimethylammonio)-1-propanesulfonate] (CHAPS), and sodium deoxycholate (SDC), with and without enzymatic digestions, which have been shown to be effective for cell removal [12].

We have recently investigated the efficacy of decellularized corneal matrix prepared by high hydrostatic pressure (HHP) [13, 14]. In our previous study, we demonstrated that the architecture and physical properties of decellularized corneal matrix were maintained, compared with that of native cornea. We also reported that decellularized corneal matrix had high biocompatibility and could become transparent after interlamellar transplantation into rabbit corneal stroma. These findings suggested the potential of decellularized corneal matrix as a corneal graft for corneal transplantation.

Our goal was to develop corneal substitutes that could restore vision comparable to donor corneal allografts, by promoting repair and regeneration of the damaged tissues with the decellularized corneal matrices. In the present study, we investigated corneal regeneration by DALK, using a decellularized corneal matrix obtained by HHP methodology.

## Materials and Methods

### Preparation of decellularized corneal matrix by the HHP method

Fresh porcine eyes were obtained from a local slaughterhouse (Tokyo Shibaura Organ, Tokyo, Japan). The corneal matrices with a thickness of 300 μm were excised from the ocular globes by delamination and washed in phosphate-buffered saline (PBS) (Invitrogen, Tokyo, Japan) containing penicillin (100 units/ml), streptomycin (100 μg/ml), and dextran (3.5% w/v, molecular weight, 70,000) (Tokyo Chemical Industry Co., Ltd., Tokyo, Japan).

Decellularized corneal matrices were prepared by the HHP method as previously described [14]. In brief, the corneal matrices were completely filled with PBS containing 3.5% dextran and then sealed in a plastic pack to prevent implosion and leakage during pressure application. They were pressurized at 980 MPa at 10°C for 10 minutes using a cold isostatic pressurization machine (Dr. CHEF; Kobe Steel, Ltd., Hyogo, Japan) to destroy the cells. Then, the corneal matrices were washed by continuous gradual shaking in an EGM-2 medium (Lonza Japan Ltd., Tokyo, Japan) containing DNase I (0.2 mg/ml) (Roche Diagnostics, Tokyo, Japan), antibiotics, and dextran at 37°C for 72 hours. The specimens were fixed in 4% paraformaldehyde at room temperature for 24 hours. They were then dehydrated stepwise using ethanol, immersed in xylene, and embedded in paraffin. Paraffin sections were stained with Mayer's hematoxylin and eosin (H-E), and immunostained with anti-α-Gal epitope antibody (clone M86; dilution; 1:25; Alexis Biochemicals Inc., Lausen, Switzerland).

### DALK

Adult Japanese white rabbits (male, 2.5–3 kg, 12 weeks old) (Kitayama Labes, Nagano, Japan) were used (n = 6). All animals were treated in accordance with the ARVO Statement on the Use of Animals in Ophthalmic and Vision Research, and all animal experiments were approved by the ethics committees for animal welfare of Tokyo Medical and Dental University and the National Institutes for Materials Science.

**Fig 1:**
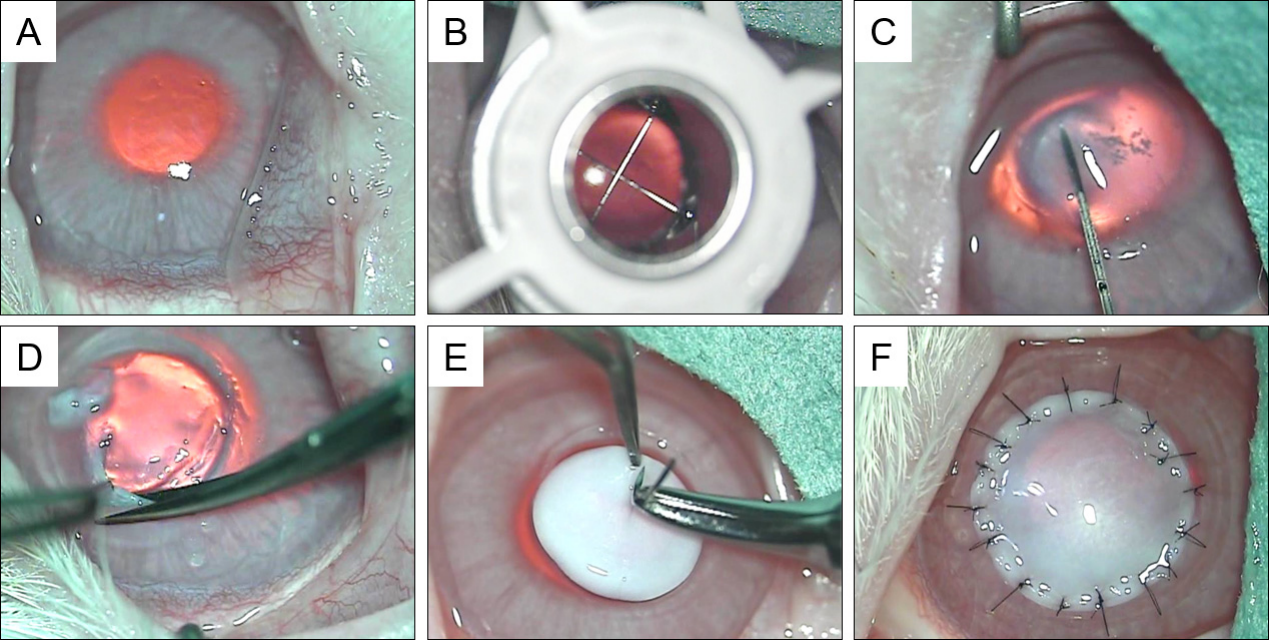
Surgical procedure of DALK using decellularized corneal matrix in a rabbit eye. (A) Normal cornea. (B) The recipient cornea was trephinated at approximately one and one fourth in depth using the 6.0 mm diameter Hessbarg-Barron vacuum trephine. (C) Hydrodelamination was performed through the perforated corneal stromal area. (D) Following the removal of stromal tissue, Descemet’s membrane was completely exposed in the transplantation area. (E) A decellularized corneal graft sutured to the recipient bed with interrupted sutures.

Recipient animals were anesthetized with sodium pentobarbital (35 mg/kg) (Somnopentyl; Kyoritsu Seiyaku Corporation, Tokyo, Japan) and topical 0.4% oxybuprocaine hydrochloride (Benoxil; Santen Pharmaceutical Co., Ltd., Osaka, Japan). Only one eye was operated on for each animal (Fig 1A). The recipient cornea was trephinated at approximately one and one fourth in depth using the 6.0 mm diameter Hessbarg-Barron vacuum trephine (JedMed Instrument Company, St. Louis, MO, USA) (Fig 1B) followed by the excision of additional stromal tissue with hydrodissection in which saline was injected into the intrastromal tissue (Fig 1C). The removal of the deep stromal tissue proceeded to the smooth surface of Descemet’s membrane (Fig 1D).

Decellularized corneas were trephinated using biopsy punches (Kai Industries Co., Ltd., Gifu, Japan) with the same size as the recipient bed. Decellularized corneal discs were then placed into the space and fixed with 16 interrupted 10–0 nylon cardinal sutures (Fig 1E and 1F). Topical steroid, 0.1% betamethasone sodium phosphate (Rinderon-A; Shionogi & Co., Ltd., Osaka, Japan), and 0.5% levofloxacin hydrate (Cravit; Daiichi Sankyo Co., Ltd., Tokyo, Japan) were instilled in the eye twice daily for 3 weeks. After 6 months, the rabbits were sacrificed with an overdose of sodium pentobarbital. The rabbit corneas, including the implants, were excised and frozen in Tissue-Tek O.C.T compound (Sakura Finetechnical Co., Ltd., Tokyo, Japan) for histological and immunohistochemical analysis.

### Biomicroscopic grading of corneal haze

Corneal haze was graded according to the haze grading system previously reported by Fantes et al [15]: grade 0, completely clear cornea; grade 0.5, a trace of haze seen with careful oblique illumination with slit lamp biomicroscopy; grade 1, more prominent haze not interfering with visibility of fine iris details; grade 2, mild obscuration of iris details; grade 3, moderate obscuration of iris details and the lens; and grade 4, complete opacification of the stroma with no visibility of iris details. Haze grading was performed in a masked manner by three independent observers.

### Fluorescein staining

Fluorescein staining was used to examine migration of the corneal epithelium into the transplanted decellularized cornea. In brief, a few drops of physiological saline were placed on a fluores ocular examination test paper (Showa Yakuhin Kako Co., Ltd., Tokyo, Japan). Then, the excess fluorescein solution was removed, and the fluores ocular examination test paper was gently applied to the lid margin. The recipient animals blinked naturally several times. The ocular surface was observed with a biomicroscope under blue light illumination. The fluorescein-stained area of the cornea was measured with an image analyser (Image J; NIH, Bethesda, MD, USA).

### Immunohistochemistry

Frozen sections were immunostained for macrophages/monocytes with anti-rabbit macrophage/monocyte monoclonal antibody (RbM2; dilution 1:25; Trans Genic Inc., Kumamoto, Japan), vascular smooth muscle cells with anti-human smooth muscle actin monoclonal antibody (1A4; dilution 1:100; Dako Japan, Tokyo, Japan), epitheliums with anti-rabbit keratin K3/K76 monoclonal antibody (AE5; dilution 1:3000; Merck Millipore, Darmstadt, Germany), and proliferating cell nuclear antigen (PCNA) with anti-human PCNA antibody (PC10; dilution 1:1200; Santa Cruz Biotechnology, Inc., TX, USA). Ten micron frozen sections were cut on a cryostat at -20°C and fixed with cold acetone for 15 minutes. Endogenous peroxidase was inactivated with 3% (v/v) hydrogen peroxide (H_2_O_2_) in PBS. The sections were incubated with primary antibody overnight at 4°C. After incubation with secondary antibody (Dako ENVISION+ system, peroxidase conjugate) at room temperature for 30 minutes and staining with 3, 3'-diaminobenzidinetetrahydrochloride (DAB), the sections were counterstained with hematoxylin. The sections were observed under a fluorescence microscope equipped with a digital camera (BZ-X700; Keyence, Tokyo, Japan).

## Results

### Corneal decellularization

**Fig 2:**
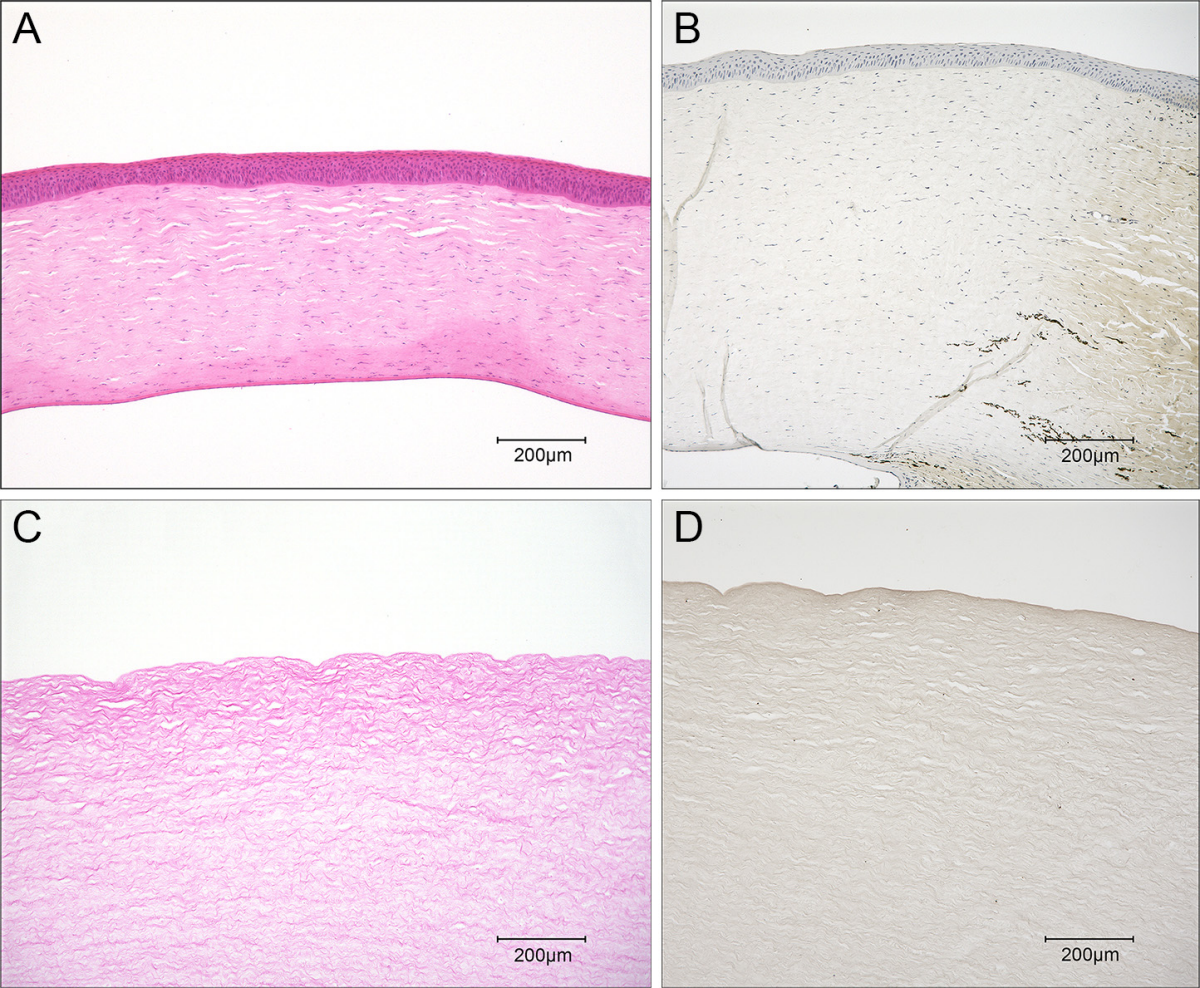
Histological images of native porcine cornea (A, B) and decellularized corneal matrix (C, D) stained with haematoxylin and eosin (H-E) and anti-α-Gal antibody, respectively. Scale bar: 200 μm.

Fig 2 shows histological images of the decellularized corneal matrix. Decellularization of the corneal matrices was achieved by high hydrostatic pressure followed by a shaking wash with EGM-2 medium. H-E staining revealed that no cellular components were observed in the decellularized corneal matrix, and the fibrillary architecture of the decellularized corneal matrix was well preserved (Fig 2A and 2C). The amount of residual DNA was less than 50ng/mg, which suggested by Crapo et al. to indicate a sufficient decellularization [16]. In addition, immunohistochemistry showed no α-Gal epitopes in the decellularized corneal matrix (Fig 2B and 2D).

### Evaluation of decellularized cornea matrix using the DALK model

**Fig 3:**
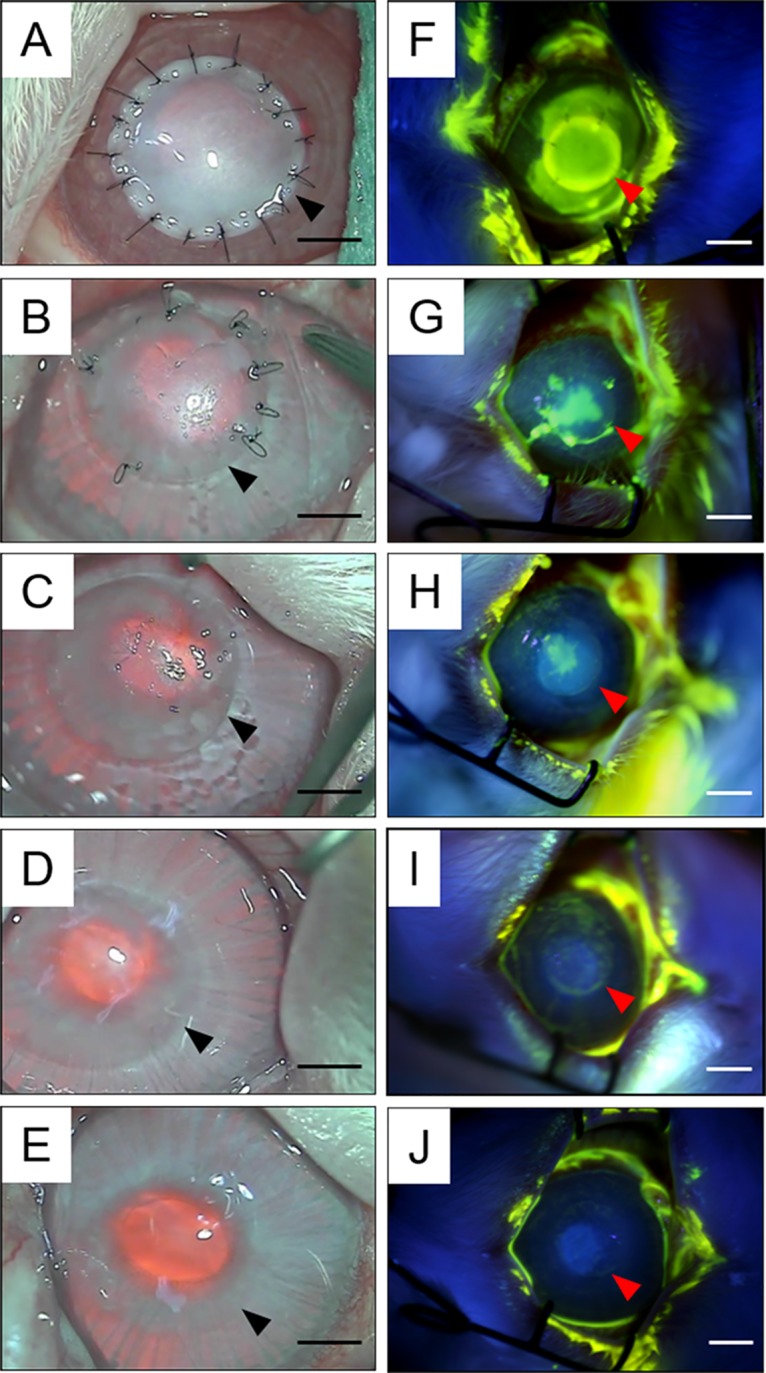
Macroscopic images and images of the rabbit eye stained with fluorescein immediately (A, F), at 2 weeks (B, G), at 1 month (C, H), at 2 months (D, I), and at 6 months (E, J) following the transplantation of decellularized corneal matrix. Arrows indicate the boundaries between decellularized corneal matrix and recipient cornea. Scale bar: 2 mm.

**Fig 4:**
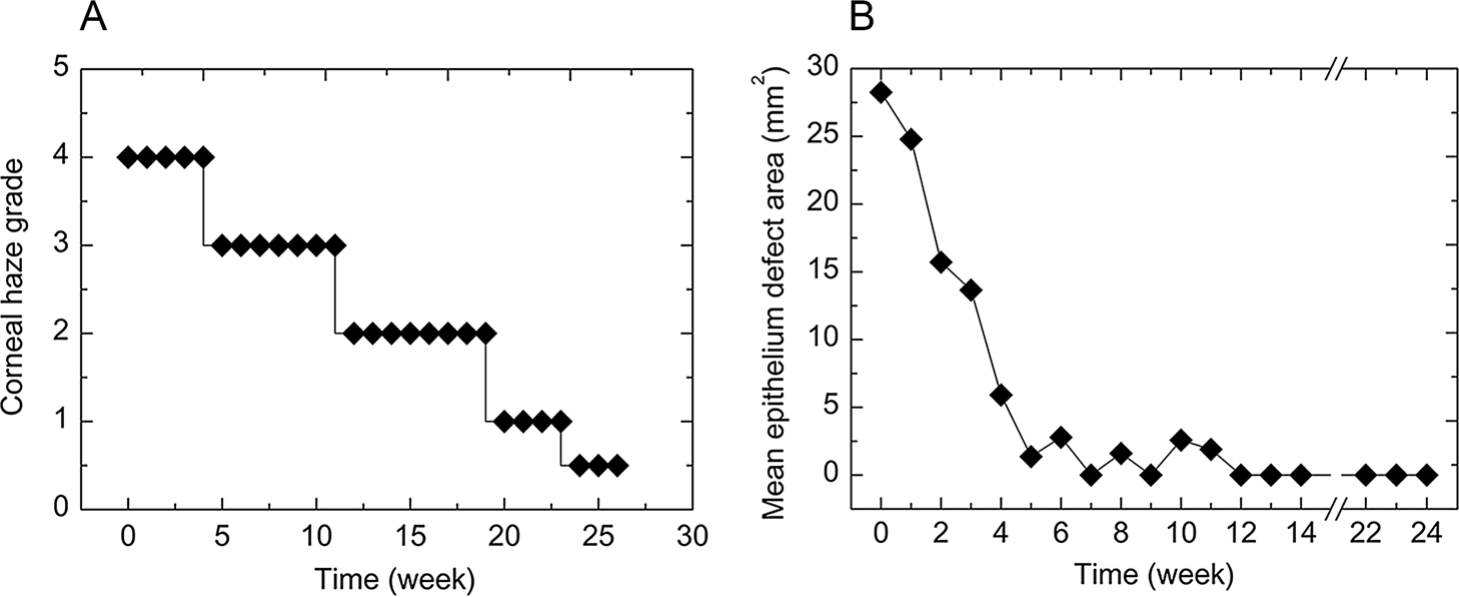
Changes in haze grading of decellularized corneal matrix after DALK over a 6-month follow-up period (A). Time course of corneal epithelial wound healing of the decellularized corneal matrix after deep anterior lamellar keratoplasty. (B). The epithelial defect areas calculated from fluorescein-stained images.

Decellularized corneal matrices were implanted into rabbit corneal stroma after deep keratectomy. The time-dependent wound healing process and haze grading of decellularized corneal matrix are shown in Figs 3 and 4, respectively. As shown in Fig 1, the decellularized corneal matrices were opaque immediately after transplantation, and corneal haze was most intense with a score of grade 4 (Figs 3A and 4A). Also, the entire graft was fluorescein dye-positive (Fig 3B). The corneal haze remained unchanged until 4 weeks, but the transparency and corneal oedema gradually improved (Fig 3D and 3E). After approximately 8 weeks after surgery, the corneal transparency significantly recovered, and the corneal haze could not be distinguished with the naked eye. The corneal epithelial defect that was fluorescein dye-positive was only slightly detectable. Subsequently, the degree of corneal haze and epithelial defect improved over time, and there was no significant difference compared with native cornea at 6 months after surgery. There were no signs of rejection including re-opacification, neovascularization within the implant or surrounding recipient corneal rim at any time until 6 months after surgery.

## Epithelial healing (re-epithelialization)

The time course of epithelial healing of the decellularized corneal matrix from a representative animal is shown in Fig 4B. The trends of epithelial healing in all grafts had a consistent pattern that involved a curvilinear exponential slope, with increasing healing latency towards complete epithelial closure. Although the initial migration of epithelial cells over the grafts was slow, corneal epithelial healing was entirely completed within 3 months. Subsequently, no corneal epithelial defect was observed until at least 6 months after surgery. No difference in quality of regenerating epithelium was found.

### Histological analysis

**Fig 5:**
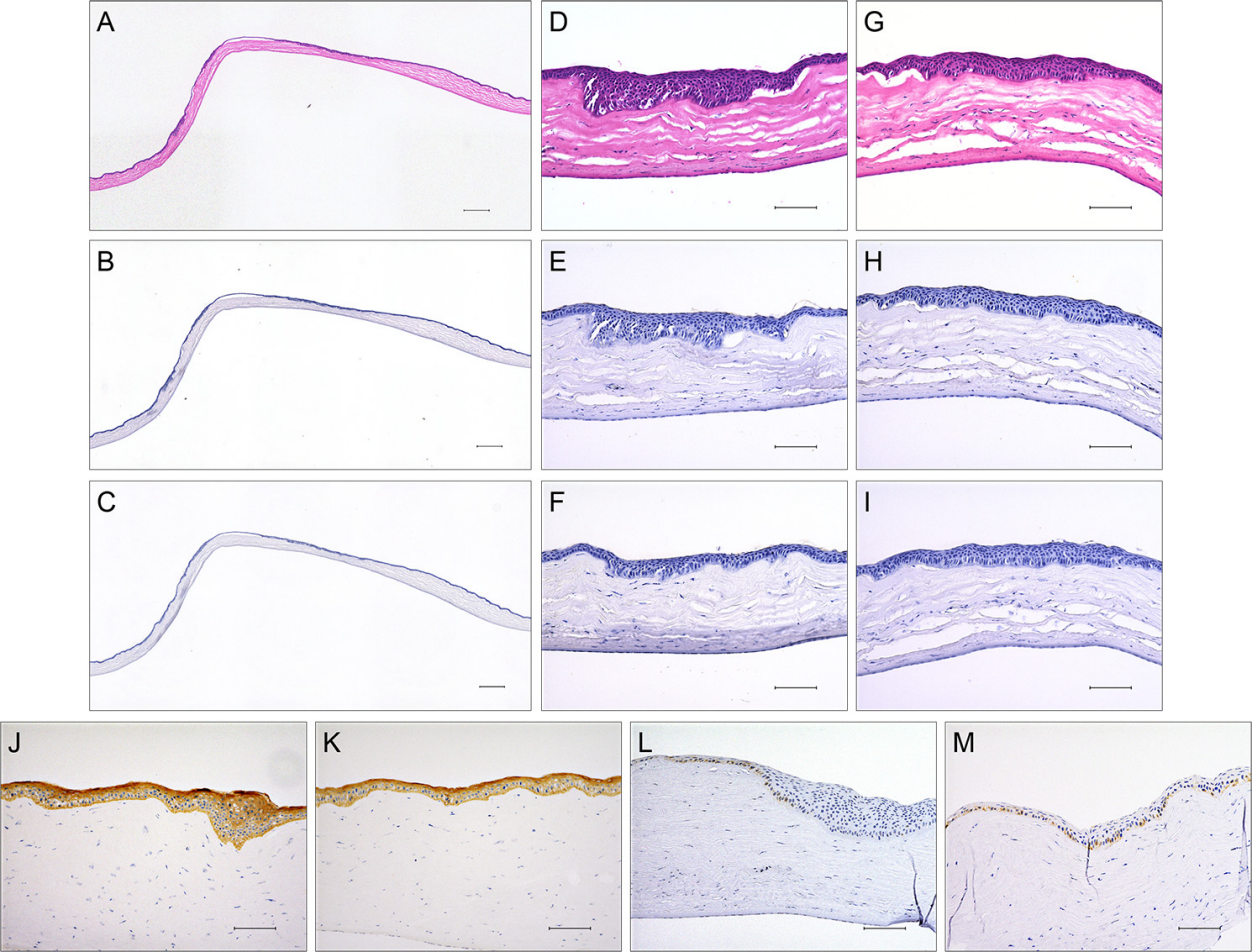
Representative histological sections of decellularized corneal matrix at 6 months after surgery. Sections were stained with H-E (A), antibody to macrophages/monocytes marker (CD68) (B), and antibody to vascular smooth muscle cells marker (α-SMA) (C). Scale bar: 500 μm (A-C). Higher magnification shows the edge (D, E, F) and centre (G, H, I) of the transplantation region. Histological images of decellularized corneal matrix stained with keratin and PCNA. (J-M). Scale bar: 100μm.

Fig 5 shows histological images of the implanted decellularized corneal matrices after 6 months of surgery. The receptive rabbit keratocytes had infiltrated into the decellularized corneal matrix. The implanted decellularized corneal matrix was completely integrated with the surrounding tissue, making it difficult to distinguish the implanted decellularized corneal matrix from native tissue (Fig 5A, 5D and 5G). The stratified corneal epithelium consisting of multiple layers regenerated on the decellularized corneal matrix, and there was no significant difference compared with the non-operated cornea. No evidence of epithelial downgrowth into the anterior chamber was observed. Hyperplasia of corneal epithelium was observed near the incision site (Fig 5J and 5K), while no expression of PCNA could be found in the hyperplasia region (Fig 5L). PCNA was expressed in the epithelial basal cells regenerated on the decellularized corneal matrix, which was almost identical to a normal cornea (Fig 5M). Immunostaining revealed that any inflammation cells such as macrophages/monocytes (Fig 5B, 5E and 5H), and any vascularization (Fig 5C, 5F and 5I), were not observed in the total implanted area.

## Discussion

Previous studies to characterize decellularized corneal matrices have used interlamellar transplantation in the rabbit model [14]. Although this surgical technique by itself may be sufficient to evaluate its biocompatibility, it does not address the possible use of decellularized corneal matrices in a clinical application. In this study, we have therefore conducted deep anterior lamellar keratoplasty (DALK) used in clinical practice, employing grafts of decellularized corneal matrices in the rabbit model.

The decellularized corneal matrices were opaque immediately after surgery, but the grade of corneal haze gradually decreased, eventually becoming transparent with a score of grade 0.5. One possible reason for the long recovery time may be that the well-organized arrangement of the collagen fibres in the stroma was damaged slightly by the decellularization treatment, and it took several months to recover its original arrangement after surgery. Another reason may involve an oedema of the decellularized corneal matrices, which may have been remedied by the pump function of the receptive corneal endothelium.

Rapid re-epithelialization of the decellularized corneal matrix through corneal epithelium migration, proliferation, and differentiation is crucially important, because it plays a prominent role against infection, ulceration, inflammation, preservation of transparency and integrity, and eventually graft failure [17]. We carefully monitored the re-epithelialization on a daily basis during the 6 months. Our results showed that the decellularized corneal matrices supported epithelial growth directly on the surface, and allowed for regeneration of a morphology involving normal stratified epithelium. However, epithelial growth did not occur as rapidly as in a normal epithelial wound, which heals in several days. Though the epithelium covered more than 90 percent of the decellularized corneal matrix regions within 1 month, complete epithelial coverage of the decellularized corneal matrix regions took longer than 2 months. The delay in epithelial healing might be the initial oedema of the decellularized corneal matrices. In general, corneal epithelial healing is known to be correlated with corneal stromal oedema. When corneal oedema occurs, corneal epithelium is difficult to regenerate on its surface. We have previously observed that the grafts cause deswelling and become transparent because of receptive corneal endothelium function. Thus, we thought that because epithelial coverage of grafts occurred with progression of deswelling, it took a longer time to completely cover the grafts, as shown by Fig 3.

One of the novel and attractive features of the present study was the remodelling of implanted decellularized corneal matrix by receptive rabbit keratocytes. A previous study reported that implanted decellularized cornea could remain in the receptive rabbit cornea, with few keratocytes infiltrating into them when using interlamellar keratoplasty [13, 14]. However, the implanted decellularized corneal matrix was completely integrated with the receptive rabbit cornea, and keratocytes infiltrated into the decellularized corneal matrix using DALK. One possible explanation for these different phenomena could be differences in surgical techniques, which involves the presence or absence of receptive corneal epithelium injury above implanted decellularized corneal matrix. Generally, the corneal wound healing process involves cytokine-mediated interactions between the epithelial cells and keratocytes of the stroma [18]. In a normal unwounded cornea, the keratocytes are almost quiescent. Following epithelial injury, disappearance of keratocytes was mediated by cytokines such as interleukin-1 (IL-1), released from the injured epithelial cells, plus platelet-derived growth factor (PDGF) observed as the first stromal response [19–21]. At the same time, IL-1 and PDGF penetrate the stroma, bind to receptors on keratocytes, and activate the quiescent keratocytes to differentiate into myofibroblasts adjacent to the acellular zone. Activated keratocytes produce growth factors such as transforming growth factor-β (TGFβ), hepatocyte growth factor (HGF), keratinocyte growth factor (KGF), collagenase, and metalloproteinase, resulting from the remodelling of stroma [22–25]. Thus, in the case of DALK with a graft of decellularized corneal matrix, decellularized corneal matrix is remodelled by a series of the abovementioned cascades. Additional study is therefore needed to determine what factors are the most important.

One of the main goals of corneal tissue engineering is to construct corneal substitutes from decellularized corneal matrices that are comparable with the human cornea. However, regarding initial transparency, the decellularized corneal matrices need to be improved. A future challenge will be to improve the processing conditions without compromising the transparency of the decellularized corneal matrix. In addition, further studies are required to develop the corneal substitutes with cell layers, especially corneal endothelium, to produce full thickness corneal transplantation. However, in the present study, we found that the decellularized corneal matrices not only could recover transparency, but also could remodel after being implanted. Our findings suggest that the decellularized corneal matrices satisfied the desired criteria for biomaterials used in corneal tissue engineering, and are consistent with the development of a full thickness corneal equivalent that will be available for clinical use.

## Conclusion

We have demonstrated that decellularized corneal matrix obtainable by HHP methodology can be used as grafts for DALK. The decellularized corneal matrix not only can improve transparency, but also can be remodelled after being implanted. No corneal epithelial down growth, angiogenesis, or immunological rejection was observed for 6 months after surgery. Together, these results provided the basis for the application of decellularized corneal matrices in the development of clinical corneal transplantation.
